# Three infection clusters related with potential pre-symptomatic transmission of coronavirus disease (COVID-19), Shanghai, China, January to February 2020

**DOI:** 10.2807/1560-7917.ES.2020.25.33.2000228

**Published:** 2020-08-20

**Authors:** Xiaohuan Gong, Wenjia Xiao, Yan Cui, Yuanping Wang, Dechuan Kong, Shenghua Mao, Yaxu Zheng, Lunhui Xiang, Lu Lu, Chenyan Jiang, Xiao Yu, Yiyi Zhu, Qiwen Fang, Hao Pan, Huanyu Wu

**Affiliations:** 1 Department of Infectious Disease Control and Prevention, Shanghai Municipal Center for Disease Control and Prevention, Shanghai, China; 2 These authors contributed equally to this work; 3 Putuo District Center for Disease Control and Prevention, Shanghai, China; 4 Pudong District Center for Disease Control and Prevention, Shanghai, China; 5 Baoshan District Center for Disease Control and Prevention, Shanghai, China; 6 Huangpu District Center for Disease Control and Prevention, Shanghai, China; 7 Shanghai Institutes of Preventive Medicine, Shanghai, China

**Keywords:** COVID-19, novel coronavirus, person-to-person transmission, pre-symptomatic transmission, incubation period, infection cluster

## Abstract

We report three clusters related with potential pre-symptomatic transmission of coronavirus disease (COVID-19) between January and February 2020 in Shanghai, China. Investigators interviewed suspected COVID-19 cases to collect epidemiological information, including demographic characteristics, illness onset, hospital visits, close contacts, activities’ trajectories between 14 days before illness onset and isolation, and exposure histories. Respiratory specimens of suspected cases were collected and tested for SARS-CoV-2 by real-time reverse-transcriptase polymerase chain reaction (rRT-PCR) assay. The interval between the onset of illness in the primary case and the last contact of the secondary case with the primary case in our report was 1 to 7 days. In Cluster 1 (five cases), illness onset in the five secondary cases was 2 to 5 days after the last contact with the primary case. In Cluster 2 (five cases) and Cluster 3 (four cases), the illness onset in secondary cases occurred prior to or on the same day as the onset in the primary cases. The study provides empirical evidence for transmission of COVID-19 during the incubation period and indicates that pre-symptomatic person-to-person transmission can occur following sufficient exposure to confirmed COVID-19 cases. The potential pre-symptomatic person-to-person transmission puts forward higher requirements for prevention and control measures.

## Background

An outbreak of pneumonia of unknown aetiology occurred in Wuhan, Hubei Province, China, in December 2019 [1,2]. The first cases were linked to exposure at Wuhan's Huanan Seafood Wholesale Market. A novel coronavirus, severe acute respiratory syndrome coronavirus 2 (SARS-CoV-2), was identified as the causing infectious agent [3] and the World Health Organization (WHO) declared a public health emergency of international concern on 30 January and a pandemic on 11 March 2020 [4,5]. As at 29 March 2020, there were 82,356 coronavirus disease 2019 (COVID-19) confirmed cases in China and 634,813 confirmed cases globally [6]. The COVID-19 pandemic has since continued to progress rapidly worldwide [7,8].

As at 21 January 2020, Hubei Province reported 375 confirmed cases and Shanghai had reported nine confirmed cases [9]. On 24 January, the Shanghai Municipal Government declared the first-level response for a major public health emergency to firmly curb the spread of the epidemic [10]. Stringent prevention and control measures were implemented, including strengthening health screening and quarantine, cancelling various large-scale public activities, encouraging people to stay at home and to wear a mask in unavoidable outside activities.

There was early evidence for human-to-human transmission among close contacts, such as in hospital, family and community settings [11-14]. Although evidence of pre-symptomatic transmission accumulated [15-17], the infectivity and duration of transmission during the incubation period have been inconclusive. These key epidemiological parameters, however, are important for outbreak control and to reduce virus transmission. Virus spread by pre-symptomatic cases poses great challenges to disease control and has an important impact on preventive strategies.

## Outbreak detection

Between 26 and 30 January 2020, three hospitals in two districts of Shanghai city reported five suspected COVID-19 cases to the local district Centers for Disease Control and Prevention (CDC). The five cases (Cluster 1) were laboratory-confirmed 1–2 days later by the Shanghai municipal CDC laboratory; two were index cases and three were close contacts, i.e. friends and close family members. Cases had histories of common activities, such as travelling and dining, or were living together.

On 1 February 2020, another hospital in Shanghai reported one suspected COVID-19 case and six of their close contacts to the local district CDC; four of the close contacts tested positive for SARS-CoV-2 by real-time reverse-transcriptase polymerase chain-reaction (rRT-PCR), 1–2 days later (Cluster 2). The seven persons were from one family, including four grandparents plus a young couple and their infant. Two grandparents had come to Shanghai from Wuhan on 21 January.

From 21–23 January 2020, a further hospital in Shanghai reported four suspected COVID-19 cases to the local district CDC. These four cases (Cluster 3), two index cases and two close contacts, were laboratory-confirmed 1 day later. They were from two couples and two of the four individuals were siblings. One couple had come to Shanghai from Wuhan on 15 January and had lived with the other couple since then.

Initial investigations revealed the epidemiological links in each of these thee clusters. Further epidemiological investigations, control measures and specimen collection were conducted by a joint field epidemiology team from the respective days when reports were received. We report the key findings of the field epidemiological investigations of the three infection clusters related with potential pre-symptomatic transmission of COVID-19.

## Methods

### Epidemiological investigation

A joint field epidemiology team, comprising public health physicians from Shanghai municipal CDC and local district CDCs, was formed and conducted detailed field investigations from the day COVID-19 case reports were received. Investigators interviewed COVID-19 cases, close contacts and healthcare workers directly (face-to-face or over the phone) to collect epidemiological information including demographic characteristics, date of illness onset, hospital visits, close contacts, activities’ trajectories between 14 days before illness onset and isolation and exposure histories (i.e. travel to or living in Wuhan or Hubei Province, visiting any other area with local sustained transmission of SARS-CoV-2, contact with persons with respiratory symptoms, contact with suspected or confirmed COVID-19 cases). In addition to interviews, medical records and travel records were checked, security cameras’ videos were retrieved, and on-site investigation of key public settings were performed. The epidemiological information of cases from multiple sources was cross-checked to ensure the reliability of information. Once an infection cluster was identified, epidemiological links and transmission chains were analysed.

### Laboratory detection

Upper respiratory specimens (nasopharyngeal swab, throat swab and/or nasopharyngeal-throat swab) and/or lower respiratory specimens (sputum) of suspected cases were collected and tested for SARS-CoV-2 by rRT-PCR assay in the Shanghai municipal CDC laboratory. The viral target included open reading frame 1ab (ORF1ab) and nucleocapsid protein (N). The specimen was positive for COVID-19 only if both viral targets were positive [18,19].

### Definitions of cases and contacts

We used the 3rd version of Prevention and Control Guidelines for Novel Coronavirus Pneumonia by the National Health Commission of the People's Republic of China‘s case definition [18]. A suspected case was defined as any person meeting clinical signs of COVID-19 and/or with epidemiological histories. A confirmed case was any suspected case with respiratory samples testing rRT-PCR-positive for SARS-CoV-2.

Epidemiological histories were defined as (i) history of travelling or residing in Wuhan or any other areas where local sustained transmission of COVID-19 existed within 14 days before illness onset, (ii) history of contact with patients with fever or respiratory symptoms from Wuhan or any other area where local sustained transmission of COVID-19 existed within 14 days before illness onset, and (iii) clustering of illness onsets, or having an epidemiological association with cases rRT-PCR-positive for SARS-CoV-2.

Clinical and laboratory signs included were (i) fever, (ii) radiological evidence of pneumonia, (iii) normal or under normal white blood cell count in early stage, or under normal lymphocyte count.

An infection cluster was identified if more than one SARS-CoV-2 rRT-PCR-positive case was found in a confined environment or group (such as a family, a company, etc.) within 14 days, and there was a possibility of interpersonal transmission because of close contact or co-exposure.

A close contact was anyone who was closely in contact with a suspected, confirmed and asymptomatic case without effective personal protection (classified protection according to the contact situation, including gloves, medical protective masks, protective face screens, isolation clothing, etc.) since onset of symptoms in the suspected case and confirmed case or the day asymptomatic case’s specimens were collected. The close contact included: (i) living, working, or studying in one house or classroom, (ii) diagnosing, treating, or visiting cases in hospital ward, (iii) being within short distance in the same vehicle, (iv) other situations assessed by the field investigators.

### Ethical statement

The epidemiological investigations were carried out according to the Law of the People's Republic of China on prevention and control of infectious diseases [20]. Ethical approval was not required because the CDCs are able to access and use personal identifiable information for infectious disease outbreak investigation according to the Law of the People's Republic of China on prevention and control of infectious diseases [20]. All cases were informed about the related rights and obligations and oral consent was obtained from all cases. Details were anonymised to protect the individual’s privacy.

## Results

### Description of clusters

#### Cluster 1

Cluster 1 involved five confirmed COVID-19 cases; three females and two males. Cases 1A to 1C, two males and one female, all in their 20s, were friends. Case 1D also in their 20s was the partner of Case 1A whereas Case 1E, who was in their 50s, was parent of Case 1D.

Cases 1A to 1D lived in Shanghai. On 12 January, Case 1D went on duty travel to a city in Jiangsu Province, accompanied by Case 1A, where Case 1A went for a haircut and to a gym for exercise. Two days later, Cases 1A and 1D travelled to a city in Anhui Province, where Case 1E lived. Here, Cases 1A and 1D participated in a wedding and then a family dinner with relatives. Case 1A exercised with Case 1D twice and exercised alone twice in Gym X. Five days after their arrival, Cases 1A, 1D and 1E returned to Shanghai. Cases 1A dined with Cases 1B, 1C and two friends in a hotpot restaurant between 17:00 and 20:00 on the same day. Then they played mah-jong in a separate room with poor ventilation in the chess and cards parlour, between 20:00 and 23:00. These five people went home separately and had no further contact with each other before illness onset.

Case 1A became symptomatic with fever (38.0 °C) at night on 20 January. They presented to hospital accompanied by a parent on 24 January and were diagnosed with bronchitis. The examination showed: body temperature was 38.5 °C, white blood cell count was 6.02 x 10^9^/L (norm 4.0–10.0 x 10^9^/L). Influenza A and B antigen tests were both negative. Case 1A visited the hospital again, accompanied by their parent, on 29 January because of persisting symptoms. Chest computed tomography (CT) scan showed scattered patches and increased density in both lungs. They went back home that day. Following Case 1E’s detection as suspected COVID-19 case on 29 January, Case 1A returned to hospital alone and was suspected as COVID-19 case one day later, when they were isolated and treated. The nasopharyngeal-throat swab was rRT-PCR positive for SARS-CoV-2 on 1 February.

Case 1B developed fever (39.0 °C) at 02:00 on 22 January. They developed headache, productive cough (bloodshot) and myalgia subsequently during 23 and 25 January. They presented to hospital accompanied by their parent on 22 January and 25 January, respectively. Influenza B antigen test was positive and influenza A antigen test was negative. On 26 January, they went to hospital again, accompanied by their parent, were suspected as COVID-19 case, were isolated and received treatment at the hospital. The examination showed: white blood cell count was 4.0 x 10^9^/L. Chest CT scan showed multiple ground glass opacities in both lungs. The nasopharyngeal-throat swab and sputum specimen were both rRT-PCR positive for SARS-CoV-2 on 27 January. Case 1B died in February at the treating hospital.

Case 1C had a fever (38.7 °C) at 09:00 on 25 January. They visited the hospital accompanied by their parent on 26 January, got symptomatic treatment and went back home. As Case 1B’s close contact with symptoms, Case 1C visited the hospital again, alone; they were suspected as COVID-19 case, they were isolated and received treatment at hospital on 29 January. The examination showed: body temperature was 37.6 °C, white blood cell count was 3.6 x 10^9^/L. Chest CT scan showed infection in the left lung. The sputum specimen was rRT-PCR positive for SARS-CoV-2 on 30 January.

**Figure 1:**
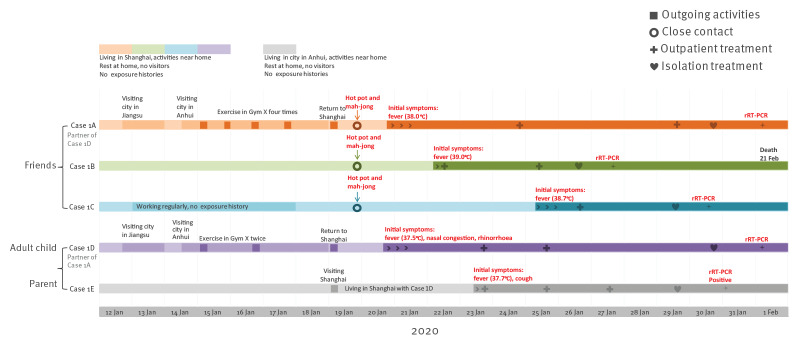
Timeline of exposure to pre-symptomatic case and illness onsets of cases in Cluster 1 of COVID-19 outbreak, Shanghai, China, 12 January–1 February 2020 (n = 5)

Cases 1D and 1E became symptomatic on 20 January and 23 January, respectively. Case 1E was isolated and received treatment on 29 January. As Case 1E’s close contact with symptoms, Case 1D was isolated and received treatment on 30 January. Respiratory specimens of Cases 1D and 1E were rRT-PCR positive for SARS-CoV-2 on 1 February and 31 January, respectively (Figure 1).

All five cases of Cluster 1 wore masks during their visits to hospital. In addition to the confirmed cases, there were seven close contacts of Cluster 1 cases: Case 1A’s parents and a close family member, as well as Case 1B and 1C’s parents. The close contacts were all living together with cases in respective households. None of them had symptoms or signs compatible with COVID-19 during the 14-day medical observation at home.

Among the five cases in Cluster 1, Cases 1A and 1D travelled outside of Shanghai, and Case 1E lived outside Shanghai. Cases 1A, 1D and 1E did not have contact with persons known to have fever or respiratory symptoms in the cities they had recently visited. During the wedding and family dinners, there were no participants from Hubei Province or any other areas with local sustained transmission of SARS-CoV-2 at the time. Gym X turned out to be the most probable source of infection of Cases 1A and 1D. From 11 January to 19 January, five persons exercised in Gym X on several occasions, who were confirmed as COVID-19 cases by the local CDC in early February. This time period overlapped with the time period when Cases 1A and 1D exercised in Gym X. None of the five cases in Cluster 1 had contact with other persons known to have fever or respiratory symptoms in Shanghai. Close contact between Cases 1A, 1B and 1C when Case 1A was asymptomatic was the likely infection source for Cases 1B and 1C.

#### Cluster 2

Cluster 2 involved five confirmed COVID-19 cases, three males and two females aged between 9 months and their early 60s. Cases 2A and 2B are parents of Case 2D and 2C, respectively. Case 2C and 2D are Case 2E’s (infant case) parents.

Case 2A and their spouse lived in Wuhan, Hubei province. They stayed at home except for purchasing food items in market. They had not been to Wuhan’s Huanan Seafood Wholesale Market or been in contact with wild animals in Wuhan. Case 2B had diabetes as underlying disease. Cases 2C, 2D and 2E lived together in an apartment in Shanghai. Case 2B lived together with their spouse in another apartment in Shanghai and their daily activities were purchasing food items at the market and cooking in Case 2C and 2D’s home. They had lunch and dinner with the young family and went back to their own home every day. Case 2C commuted to and from work regularly. Case 2D took care of Case 2E at home.

Case 2A and spouse drove from Wuhan to Shanghai on the morning of 21 January and lived in Case 2C and 2D’s apartment since then. Case 2B and their spouse arrived in Case 2C’s apartment soon afterwards. Case 2B went back home after having a conversation with Case 2A and their spouse for ca 1.5 hours. Case 2A, their spouse, Case 2B’s spouse and Case 2D talked to each other, had lunch and had dinner together until evening. Case 2C joined dinner after work in the evening. Case 2B’s spouse went back home that evening. On 22 January and 23 January, Case 2B and their spouse stayed at their own apartment. At 09:30 on 24 January, Case 2B and their spouse arrived in Case 2C and 2D’s apartment. The seven persons stayed together, had lunch and dinner, celebrating the spring festival until almost 19:00 that evening. Case 2B and their spouse went back home that evening. After 24 January, Case 2B and their spouse stayed at their own apartment and did not go outside. After 24 January, Case 2A and their spouse, Cases 2C, 2D and 2E stayed at Cases 2C and 2D’s apartment and did not go outside.

Case 2A developed cough around noon of 31 January. As Case 2B’s close contact with symptoms, they presented to hospital, were suspected as COVID-19 case, and were admitted to hospital for isolation and treatment on 2 February. The nasopharyngeal swab was rRT-PCR positive for SARS-CoV-2 on 3 February.

Case 2B developed fever (38.0 °C) at 19:00 at their own home on 24 January. They presented to hospital on 25 January and 30 January, twice, wearing mask, accompanied by their spouse. Because Case 2B had fever, they visited hospital again, accompanied by their spouse, were suspected as COVID-19 case, and were admitted to hospital for isolation and treatment on 1 February. The examination showed: body temperature was 37.3 °C, white blood cell count was 3.9 x 10^9^/L. Chest CT scan showed infectious lesions of the upper lobe of both lungs and thin nodular shadow of the upper lobe of the left lung. The nasopharyngeal swab was rRT-PCR positive for SARS-CoV-2 on 2 February.

Case 2C became symptomatic with a light productive cough and diarrhoea around noon of 31 January, 1 hour after illness onset of Case 2A. As Case 2B’s close contact with symptoms, they presented to hospital, were suspected as COVID-19 case, were isolated and received treatment on 2 February. The nasopharyngeal-throat swabs were rRT-PCR negative for SARS-CoV-2 on 3 February and positive on 4 February.

**Figure 2:**
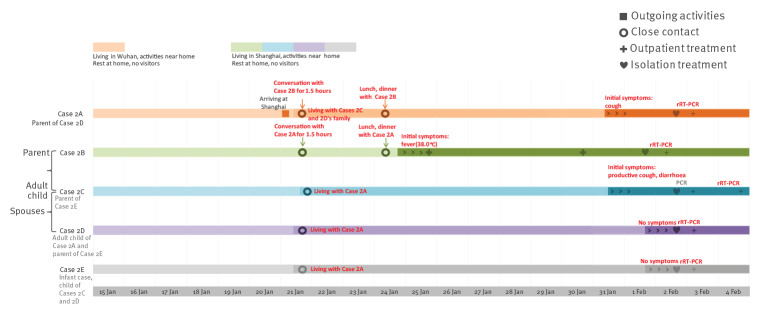
Timeline of exposure to pre-symptomatic case and illness onsets of cases in Cluster 2 of COVID-19 outbreak, Shanghai, China, 15 January–4 February 2020 (n = 5)

As Case 2B’s close contacts, Cases 2A and 2B’s spouses, as well as Cases 2D and 2E had been asymptomatic and were admitted to hospital on 2 February. The nasopharyngeal swabs of cases 2D and 2E were both rRT-PCR positive for SARS-CoV-2 on 3 February. The nasopharyngeal swabs of Cases 2A and 2B’s spouses were both twice rRT-PCR negative for SARS-CoV-2 at an interval of 24 hours (Figure 2).

Except for confirmed cases and Cases 2A and 2B’s spouses, there were no other close contacts of Cluster 2.

Among the five cases in Cluster 2, only Case 2A had a history of living in Wuhan. Cases 2B, 2C, 2D and 2E had not travelled outside Shanghai. In Shanghai, none of these five cases had known contact with other persons with fever or respiratory symptoms and other persons coming from Hubei Province or any other areas where local sustained transmission of SARS-CoV-2 existed. Before Case 2B’s onset of symptoms, Case 2B went to a market and Case 2C went to work regularly. Considering the COVID-19 situation in Shanghai and Wuhan at that time, close contact with pre-symptomatic Case 2A was the most likely infection source of Cases 2B and 2C. The likelihood of Cases 2B and 2C’s exposure to other sources in Shanghai was considered much lower.

#### Cluster 3

Cluster 3 involved four confirmed COVID-19 cases, two males and two females, aged between 60 and 80 years. Case 3A is Case 3D’s spouse. Case 3C is Case 3B’s spouse. Case 3A and Case 3C are siblings.

Cases 3A and 3D lived in Wuhan, Hubei Province. They stayed at home except for purchasing food items in the market. They had not been to Wuhan’s Huanan Seafood Wholesale Market or been in contact with wild animals in Wuhan. Cases 3B and 3C lived in Shanghai. Case 3B was a long-term bedridden patient with several comorbidities (hypertension, cardiac disease and chronic obstructive pulmonary disease). Case 3C purchased food items, cooked meals and took care of Case 3B at home.

Cases 3A and 3D arrived in Shanghai by train on 15 January and stayed in Case 3B and 3C’s apartment since 16:00 that day. On 16 January and 17 January, Cases 3A, 3C and 3D went shopping at a nearby supermarket twice. Apart from this, the four cases stayed at home and did not go outside.

Case 3A became symptomatic with chills and fever at 22:00 on 20 January. They presented to hospital, were suspected as COVID-19 case, and were admitted to hospital for isolation and treatment on 21 January. The examination showed: body temperature was 38.5 °C, white blood cell count was 4.37 x 10^9^/L. Chest CT scan showed two patchy ground glass opacity high-density shadows in the right lung. The throat swab was rRT-PCR positive for SARS-CoV-2 on 22 January. Case 3A died in hospital in March.

Case 3B developed poor appetite and dry cough on the morning of 20 January and fever (38.2 °C) on 21 January. They presented to hospital, were suspected as COVID-19 case, and admitted to hospital for isolation and treatment on 21 January. The examination showed: white blood cell count was 7.16 x 10^9^/L. Influenza A and B antigen tests were both negative. Chest CT scan showed interstitial hyperplasia and infection of both lungs. The nasopharyngeal swab and throat swab were both rRT-PCR positive for SARS-CoV-2 on 22 January. Case 3B died in hospital on 25 January.

**Figure 3:**
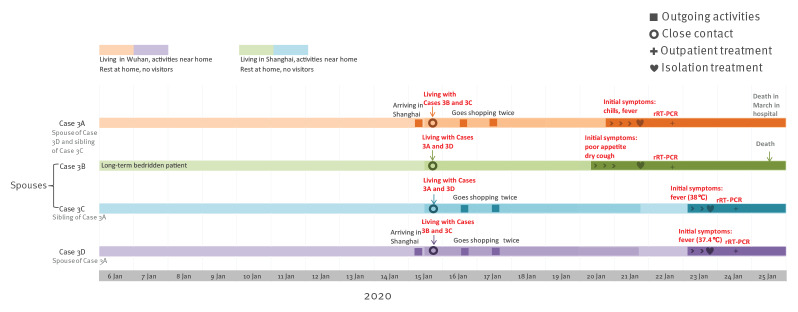
Timeline of exposure to pre-symptomatic case and illness onsets of cases in Cluster 3 of COVID-19 outbreak, Shanghai, China, 6 January–25 January 2020 (n = 4)

As close contact of Cases 3A and 3B, Case 3C developed fever (38.2 °C); Case 3D also developed fever (37.4 °C), both at 09:00 on 23 January, and they were both admitted to hospital on the same day. The nasopharyngeal swabs and sputum specimens of Cases 3C and 3D were both rRT-PCR positive for SARS-CoV-2 on 24 January (Figure 3).

Except for confirmed cases, there were three close contacts of Cluster 3 cases, including Case 3B and 3C‘s child, their spouse and grandchild, who had visited Case 3B. They did not have any symptoms or signs during the 14-day medical observation at home.

Cases 3A and 3D had histories of living in Wuhan. Cases 3B and 3C had not travelled outside Shanghai. In Shanghai, none of the four cases had known contact with other persons with fever or respiratory symptoms and persons coming from Hubei Province or any other areas where local sustained transmission of COVID-19 existed. Case 3B was a long-term bedridden patient. Close contact with Case 3A when they were pre-symptomatic was the most likely infection source of Case 3B.

### Analysis of clusters

In these three infection clusters, 14 confirmed cases developed symptoms and visited hospitals when they were in Shanghai and eight of these confirmed cases were related with potential pre-symptomatic transmission (Table 1). CDCs’ public health physicians conducted field epidemiological investigations and communicated with the cases directly; contact situations of three infection clusters are shown in Table 2. In each of the clusters, the primary cases were identified (Cases 1A, 2A, 3A) and in total five cases (Case 1B, 1C, 2B, 2C, 3B) were secondary cases who got infected by being in close contact with the primary cases. The primary cases 1A, 2A and 3A, had no clinical symptoms or signs when they were in contact with these five secondary cases, and there were no contacts after the illness onset of the primary cases. In Cluster 1, illness onset in the five secondary cases was 2 to 5 days after the last contact with the primary case. In Cluster 2 and Cluster 3, illness onset in secondary cases occurred prior to or on the same day as the onset in the primary cases. The interval between the onset of illness in the primary case and the last contact of the secondary case with the primary case in our report was 1 to 7 days. No other relevant exposure histories of the secondary cases were found.

**Table 1:** Demographic and clinical characteristics of confirmed COVID-19 cases related with potential pre-symptomatic transmission in three infection clusters, Shanghai, China, January–February 2020 (n = 8)

Cluster	Case	Age (years)	BMI	Smoking	Comorbidity	Fever (°C)	Other respiratory symptoms	Influenza antigen test	White blood cell (x 10^9^/L, (norm 4.0–10.0)	Chest computed tomography scan	Clinical outcome
Cluster 1	Case 1A	20–30	30.4	No	None	38.0–38.5	NA	Influenza A and B negative	4.1–6.0	Scattered patches and increased density in both lungs	Recovery
	Case 1B	20–30	35.5	No	None	37.6–39.0	Headache, productive cough, myalgia	Influenza B positive	4.0	Multiple ground glass opacities in both lungs	Death
	Case 1C	20–30	22.9	No	None	37.3–38.7	NA	NA	3.6	Infection in left lung	Recovery
Cluster 2	Case 2A	50–60	NA	Yes	None	NA	Cough	NA	NA	NA	Recovery
	Case 2B	60–70	22.6	Yes	Diabetes mellitus	37.3–38	NA	NA	3.8–4.2	Infectious lesions of both lungs and thin nodular shadow of left lung	Recovery
	Case 2C	30–40	28.7	No	None	37.7	Light productive cough, diarrhoea	NA	NA	NA	Recovery
Cluster 3	Case 3A	60–70	19.7	No	None	37.6–38.5	Chills	NA	4.4	Two patchy ground glass opacity high-density shadows in right lung	Death
	Case 3B	80–90	19.8	No	Hypertension, cardiac disease, COPD	38.2	Poor appetite, dry cough	Influenza A and B negative	7.2	Interstitial hyperplasia and infection of both lungs	Death

BMI: body mass index; COPD: chronic obstructive pulmonary disease; COVID-19: coronavirus disease; NA: not available.

**Table 2:** Contact situations of three infection clusters related with potential pre-symptomatic transmission, Shanghai, China, January–February 2020

Cluster	Case	Residence city	Close contact with primary cases (Cases 1A, 2A, 3A)								Time of onset of illness
			Type of contact	Persons	Duration (hours)	Contact distance (meters)	Contact frequency	Mask wearing	Surroundings	Contact period	
Cluster 1	Case 1A	Shanghai	NA	NA	NA	NA	NA	NA	NA	NA	20 Jan; night
	Case 1B	Shanghai	Hot pot dinner, mah-jong game	5	6	< 1	Frequently within 6 hours	None	Inside a restaurant, a poorly ventilated room	19 Jan; 17:00 – 23:00	22 Jan; 02:00
	Case 1C	Shanghai	Hot pot dinner, mah-jong game	5	6	< 1	Frequently within 6 hours	None			25 Jan; 09:00
Cluster 2	Case 2A	Wuhan	NA	NA	NA	NA	NA	NA	NA	NA	31 Jan; noon
	Case 2B	Shanghai	Conversation / lunch and dinner	7	11.5	< 1	Commonly / frequently	None	In 2C and 2D’s apartment	21 Jan; 09:30 – 11:00 24 Jan; 09:30 –19:00	24 Jan; 19:00
	Case 2C	Shanghai	Living together with Case 2A	5	241	< 1	Frequently	None	In 2C and 2D’s apartment	21 Jan; night until 2 Feb (isolation)	31 Jan; noon
Cluster 3	Case 3A	Wuhan	NA	NA	NA	NA	NA	NA	NA	NA	20 Jan; 22:00
	Case 3B	Shanghai	Living together with Case 3A	4	114	< 1	Frequently	None	In their own apartment	15 Jan; 16:00 until 21 Jan (isolation)	20 Jan; morning

NA: not available.

## Outbreak control measures

Multiple control measures were implemented immediately once these three clusters were detected. First, isolation and treatment was performed immediately when patients were suspected to have COVID-19 according to doctors’ judgment based on the existing guidelines [18]. Suspected cases were transferred by ambulance to a municipal-designated hospital once their respiratory specimens were rRT-PCR positive for SARS-CoV-2. Second, close contacts were put under centralised or home medical observation for 14 days since the last day of contact with cases, under supervision of a team including clinical physicians, nurses, CDC physicians, and community workers. During observation, body temperature and respiratory symptoms or signs were recorded twice every day. Third, disinfection measures were implemented in cases’ homes, visited hospitals, work places and other places where cases had spent time, to prevent secondary infections. Fourth, surveillance in fever clinics and health education for the population were strengthened especially in areas where the infection clusters occurred. Fifth, people arriving to Shanghai from other provinces or foreign countries were health quarantined for 14 days in a centralised isolation location, and observed medically with body temperature and respiratory symptoms or signs being recorded twice every day.

## Discussion

The study provides empirical evidence for transmission of COVID-19 in the pre-symptomatic phase. It supports the 5th version of Prevention and Control Guidelines for Novel Coronavirus Pneumonia published by the National Health Commission of the People's Republic of China on 21 February [21] which refers to close contacts as those in close contact with cases without effective protection from 2 days before the onset of symptoms. In their research, Zou et al. showed that the viral load detected in asymptomatic COVID-19 cases was similar to that in symptomatic ones [22], which suggests the transmission potential of asymptomatic or minimally symptomatic patients.

In April 2020, WHO interim guidelines also suggested that individuals who had contact with a confirmed case from 2 days before symptom onset should be identified and traced [23]. These changes to the earlier WHO interim guidelines emphasised the importance of looking for contacts in their pre-symptomatic stage. The longest interval between the onset of illness in the primary case and the last contact of the secondary case with the primary case in our report was 7 days, which was longer than 2 days and within the ranges of published mean incubation period (5.1–11.5 days) according to recent research [11,24,25]. An alternative explanation could be that the initial symptoms of the primary case of Cluster 2 were too mild to self-recognise. Both explanations of this study provide clues for further research on pre-symptomatic transmission of COVID-19.

Unlike SARS-CoV-1, where almost all onward transmissions occur after symptom onset [26], published evidence of pre-symptomatic transmission has been accumulating for SARS-CoV-2 [14-16,27,28]. Transmission before symptom onset has a marked effect on control and prevention of infectious diseases. It increases the probability for the population to get infected, and weakens the power of isolation because contacts may have got infected already before isolation of the cases [29]. In the study by Mizumoto et al., the estimated asymptomatic proportion was 17.9% (95% credible interval: 15.5–20.2%) [27]. The clinical spectrum and infection spectrum of COVID-19 still need to be studied deeper to help public health decision making.

Among the three infection clusters, pre-symptomatic transmission appeared to take place when (i) the exposure time was sufficiently long i.e. equal to or more than 6 hours, (ii) the exposure distance was short i.e. less than 1 m, (iii) the exposure frequency was high and the distance was short i.e. living together in one house, dining or playing together at one table, and (iv) no masks were worn when in contact. This indicates that pre-symptomatic person-to-person transmission can happen when there is sufficient exposure with a confirmed COVID-19 case. However, we do not know whether shorter or less intense exposures to pre-symptomatic cases might also lead to transmission.

There are two main limitations that need to be acknowledged. First, the evidence case reports provide is less persuasive than results of well-designed studies where information is obtained following a specific protocol. Second, even with detailed field investigation and information that was cross-checked from multiple sources, considering recall bias, there is still chance that not every possibility for transmission was recorded, such as whether there were alternative sources for Cluster 2.

This report also showed that COVID-19 can be transmitted between families, friends and cities. Transmission has taken place all over the world [30,31]. Strict measures were adopted in the early stage of the COVID-19 epidemic in Shanghai, which resulted in decreasing numbers of reported confirmed and suspected cases. In the past months people have been returning to Shanghai for work from all over China and people have been arriving in Shanghai from all over the world; Shanghai is facing great challenges in preventing imported cases. Medical observation and centralised isolation of people from abroad was strengthened. Health quarantine for 14 days in centralised isolation location for every traveller returning from other countries is crucial in preventing imported COVID-19 cases, which can lead to imported cases in this pandemic. The potential pre-symptomatic person-to-person transmission puts forward higher requirements for research and prevention and control measures. Until the infectivity and duration of incubation period transmission are conclusive, more research is needed for optimising prevention and control strategies, including seroprevalence studies, natural history studies based on population in epidemic areas, and studies about efficiency of asymptomatic transmission. The incubation period should be taken into consideration in epidemiological investigations and the identification of close contacts. Moreover, the importance of pre-symptomatic transmission in outbreak evolution needs to be in far wider and deeper consideration.
